# Syphilis about the Mouth

**Published:** 1881-05

**Authors:** F. Newland Pedley


### ARTICLE II.

### *Syphilis About the Mouth.*

BY F. NEWLAND PEDLEY, L. D. S.

A paper read before the Students' Society of the Dental Hospital of London, December 13th, 1880.

*Mr. President and Gentlemen.*—Syphilis is a subject upon which we have had no paper for some years. Yet it is a disease that is constantly exhibiting itself in the mouths of our patients, and exposing us and others to the chance of contagion by inoculation.

I regret to say that a thorough knowledge of the contagiousness of this disease is not as general amongst members of our profession as it should be, and I shall think that a good result has been achieved this evening if I induce you to take extreme care that neither operator nor patient be needlessly exposed to the inoculation of this horrible infection.

No one knows how old syphilis is, but it seems to have been well known to the Moors at the siege of Granada. The first description that we get of the disease in Europe comes from Barcelona in 1494. The physicians at that date called it Morbus Gallicus, and said that it came from France, where it was commonly known as the disease of St. Semente. A Messinese traveler, writing from Barcelona on June 18th, 1494, says :—" On arriving at this port, which is a flourishing city in Spain, I really shuddered at the sight of so many victims of contagious disease." He describes it from report, as "lasting for a year, beginning in the private parts, and then breaking out in other parts of the body." After this it gradually became a recognized and familiar disease, celebrated for its virulent and persistent character.

You are aware that under the head of syphilis are often included two diseases that are locally characterized, the one by soft sores, and the other by a hard sore. I shall make no further reference to soft sores, they are merely local, and do not affect the constitution. In speaking of syphilis to-night I allude to that malady which follows the indurated sore or Hunterian chancre.

The accepted history of a hard chancre is that a vesicle may form within an interval of from five days to a month after exposure to contagion. The vesicle cracks, breaks down, and becomes excavated, and the characteristic induration of the base occurs, feeling to the touch as if the margin of the sore were formed of a layer of parchment.

If left alone the sore will generally heal in six weeks, and about that period secondary symptoms, consisting of a coppery rash, will appear on the body, accompanied with sore throat, and possibly psoriasis about the soles and palms. The disease, if neglected, may go on to the tertiary stage, characterized by gummata and ulceration.

This hurried outline must suffice for an introduction to my true subject, which relates to the disease as seen by dentists. The commonist manifestation of it which I have noticed has been in the form of cracks, fissures, and ulcers about the tongue. There are two forms of these ulcers, *i. e.*, superficial and deep. Paget says that the former are related to the syphilitic psoriasis which appears on the palms and soles; the latter to the deep ulcers that follow gummata. Of the superficial ulcers the most common are such as form at the sides of the tongue. Some of them may not deserve the name of ulcers, for they have thin coverings of epithelium, and do not bleed when roughly touched. They may be like little starred or oblique fissures at the edge and top of the tongue, or may appear as pale, bald, raw patches on the mucous membrane, or, again, they may be single, flat-based, defined, and thin-bordered ulcers through nearly the whole thickness of the mucous membrane. With any of these forms of disease there may be round the ulcers little groups of florid papillæ. All the superficial syphilitic ulcers of the tongue are very sensitive and sore. In course of time nearly the whole surface of the tongue may become opaque, whitish, and smooth, and the whole organ may become large and thick.

In distinguishing syphilitic induration of the tongue from that due to malignant disease, Paget lays great stress upon the comparative hardness of the tumor. He says that the softness of the tumor, the presence of white stains, and the coexistence of other veneral symptoms, are to be our guide in diagnosing syphilis of the tongue.

A useful diagnostic sign by which one may distinguish syphilitic from cancerous enlargement of the tongue is that syphilitic indurations of the tongue never involve other than the lingual substance, but malignant growths usually make their way into the surrounding tissues.

The peculiar white markings which are so commonly found on the mucous membrane of the mouth of those suffering from constitutional syphilis, though not confined to that disease, are yet very valuable indications of the probable nature of the affection they accompany.

They are seldom seen in cancer, although the tongue is frequently covered with a very thin fur, which, when distributed in patches, might easily be mistaken for these white markings. The attempt to scrape it off will, however, at once distinguish between them, for the syphilitic stains are in the epithelium, and not upon it, and cannot be removed. Occasionally punctiform, they more commonly occur as broad flat patches much resembling thin films of bluish milk, at other times they are slightly raised and of a dead white color. When occurring on the back of the pharynx they have been compared by the Irish surgeons to the slimy tracks left by snails.

The deep syphilitic ulcer is usually the result of a broken-down gumma. Gummata appears in tertia syphilis. They are, at first, hard, rounded, inflammatory growths; they may form in any tissue of the body, and are exceedingly prone to break down and to leave an ulcerated surface. We often see them in the median raphe of the tongue. The typical syphilitic ulcer is described by Holmes as forming a large excavation with foul, raised, crescent edges and sloughy surface.

By a process strictly analogous to the formation of an ulcer by the breaking down of a gumma, we observe the necrotic perforation of certain bones about the mouth. The bones most liable to this form of necrosis are such as are compact in structure and have thin, soft coverings, such as the palate bones, the palate process of the superior maxilla, the vomer, etc.

It is sometimes very difficult to distinguish the deep syphilitic ulcers from the irritable, the dyspeptic, or the cancerous form. All these ulcers resemble one another greatly in shape, and it must be remembered that any sore place about the mouth may take on the specific form, provided the patient be syphilitic, hence an irritable ulcer may become specific. There are, however, certain useful points to aid our diagnosis.

*Irritable ulcers* get well when the offending tooth or irritating cause is removed. They are usually seen at the point where the teeth touch the side of the tongue or the cheeks.

*Dyspeptic ulcers* are found in the middle line of the tongue, and the aspect of that organ shows that the stomach is out of order. When the digestion is improved, the ulcers gets well.

*Syphilitic ulcers* will not get well without antisyphilitic remedies. There may be the history of the case or other evidences of syphilis to guide us.

*Cancerous ulcers* do not get well at all. The submaxillary lymphatic glands become enlarged after a time, while in tertiary syphilis it is the posterior cervical glands that are swollen.

A course of iodide of potassium settles the question between the two latter forms of ulcers, for the syphilitic ulcer heals under its influence, whilst the cancerous does not.

Another form in which syphilis about the mouth is brought before our notice is the shape of the mucous tubercle. These are described as occurring in flat, raised, oval patches generally situated at or near the junction of the mucous membrane with the skin, covered with a whitish velvety epithelial tissue. In the same situation condylomata are found, consisting of small masses of the hypertrophied structures of the skin, discharging a foul secretion.

I think we should do well to regard with extreme suspicion all cracks, fissures, and sores of any kind whatever about the lips; for, certainly it is that the secretion from these syphilitic sores is inoculable and may be conveyed from the lips of one patient to those of another, unless extreme caution be observed in thoroughly cleansing all our instruments.

Besides syphilis thus acquired by inoculation, there is the hereditary form of the disease, which runs a course very similar to that in adults. Syphilitic children go through the primary stages in utero, and can impart the affection to the healthy mother; their secondary symptoms commences usually about a month after birth, and tertiary symptoms, in the form of diseases of the eye and ear, may follow after a lapse of time.

Dentists ought to know about infantile syphilis, for it modifies the shape of the teeth; so does the mercury that is taken as a remedy for the complaint. The temporary upper central and lateral incisors are generally lost very early, and the children may be brought to us with exfoliation of particles of the alveolar process corresponding to those teeth. You will easily recognize the children by their miserable stunted look, the puny hand, protuborant forehead, flattened nose and puckered mouth. You will have no need to search for the coppery eruption on the genitals, palms of hands, and soles of feet, in order to interpret the ancient, careworn, superannuated look of the child. The treatment is mercury. Perhaps the very fact that mercury is the only cure for infantile syphilis is the cause of the strange blunder that has existed until the last few years of confounding Hutchinson's sypnilitic teeth with "mercurial teeth."

Personally, I would include all the "mercurial," "stomatitic," and "strumous" teeth under the one generic term "stomatitic;" for it is proved beyond cavil that whatever leads to even congestion of the mucous membrane of the mouth or alveolus while the tooth is in process of formation, will produce rocky, honeycombed, pitted teeth. But honeycombed teeth are not necessarily mercurial, for Mr. Samuel Cartwright says he has seen as many in Germany, where mercury is comparatively seldom given, as in this country. It is no argument in favor of the identity of

"mercurial" and syphilitic teeth that both varieties are occasionally found in the same mouth, for the "mercurial pitting" is caused by congestion of the mucous membrane, resulting from the mercury given as a remedy for infantile syphilis.

I fully accept Mr. Jonathan Hutchinson's view on the subject, and this paper would be strangely incomplete if I omitted a brief description of Hutchinson's incisors and Moon's molars. The first molar that bears Mr. Moon's name is dome-shaped and stunted, and is in itself pathognomonic of hereditary syphilis; but the teeth to which the greatest amount of attention has been directed are the permanent upper incisors. Mr. Hutchinson says: "The upper central incisors are short and narrow, their angles rounded off, and their edges exhibit a broad shallow notch." The single broad notch, of greater or less degree of depth, is hardly ever wanting. The teeth are always of bad color. On looking carefully at the surface of the notch there is almost always evidence of wearing, that is, the enamel is not perfect at the scooped-out border of the tooth. I believe that these teeth rarely present the notch at the time of their being cut, there being usually a small projecting lobe. Above the base of this lobe, which is very thin, extends a crescentic line, marking out the size that the notch will have when, as is quickly the case, the thin central plate has been broken or worn away.

The upper lateral incisors deviate but little from the natural type. Rarely are they notched, but they are often small and ill-formed.

In syphilitic mouths the canines, as are a rule, show blunted extremities, in the centres of which are seen single little tubercles. It is almost always present in syphilis, but is not a reliable diagnostic sign.

The lower incisors hardly ever show notches in their borders. Sometimes their central lobes are peculiarly elevated, and by an excessive development of their tubercles and serrations they come to resemble the teeth of fishes. Eventually the teeth become shortened and peg-shaped.

Syphilitic teeth are further distinguished from stomatitic teeth by the fact that the former look soft, worn and rounded; the latter are harder, rugged, with well defined margins.

Hutchinson's incisors are not due to mercury, for they have appeared in the mouths of children who have not taken mercury, and those who have taken mercury never displayed the characteristic syphilitic incisor.

I shall conclude by mentioning a rare form of dental caries that seems to be syphilitic. In vol. xlv, of "Medico-Chirurgical Transactions," a case is quoted by Dr. Marston of a syphilitic man whose teeth decay in a most characteristic manner:

"A dark spot would appear on the front aspect of the enamel close to the gum. The lateral incisors of the upper jaw were first affected, and disease of the remaining teeth speedily followed. The discolored spot became the seat of caries, and a minute circular hole resulted, situated in the middle line of the tooth bordering upon the gum. The disease in each tooth gradually advanced from before backwards, extending laterally at the same time, and making its way in a very definite manner till the line of caries passed through the tooth, and severed it at its junction with the fang. The lower teeth were affected in a similar way."

Dr. Marston says he has seen this very disease of the teeth follow the same course in two other cases.—*British Journal of Dental Science.*

---

